# Shortwave Radiation Calculation for Forest Plots Using Airborne LiDAR Data and Computer Graphics

**DOI:** 10.34133/2022/9856739

**Published:** 2022-07-16

**Authors:** Xinbo Xue, Shichao Jin, Feng An, Huaiqing Zhang, Jiangchuan Fan, Markus P. Eichhorn, Chengye Jin, Bangqian Chen, Ling Jiang, Ting Yun

**Affiliations:** ^1^School of Information Science and Technology, Nanjing Forestry University, Nanjing 210037, China; ^2^Forestry College, Nanjing Forestry University, Nanjing 210037, China; ^3^Plant Phenomics Research Centre, Academy for Advanced Interdisciplinary Studies, Collaborative Innovation Centre for Modern Crop Production Cosponsored by Province and Ministry, Nanjing Agricultural University, Nanjing 210095, China; ^4^Chinese Academy of Tropical Agricultural Sciences, Ministry of Agriculture, Rubber Research Institute, Danzhou Investigation and Experiment Station of Tropical Crops, Danzhou, China; ^5^Research Institute of Forestry Resource Information Techniques, Chinese Academy of Forestry, Beijing 100091, China; ^6^National Engineering Research Center for Information Technology in Agriculture, Beijing 100097, China; ^7^School of Biological, Earth and Environmental Sciences, University College Cork, Distillery Fields, North Mall, Cork T23 N73K, Ireland; ^8^Environmental Research Institute, University College Cork, Lee Road, Cork T23 XE10, Ireland

## Abstract

Forested environments feature a highly complex radiation regime, and solar radiation is hindered from penetrating into the forest by the 3D canopy structure; hence, canopy shortwave radiation varies spatiotemporally, seasonally, and meteorologically, making the radiant flux challenging to both measure and model. Here, we developed a synergetic method using airborne LiDAR data and computer graphics to model the forest canopy and calculate the radiant fluxes of three forest plots (conifer, broadleaf, and mixed). Directional incident solar beams were emitted according to the solar altitude and azimuth angles, and the forest canopy surface was decomposed into triangular elements. A ray tracing algorithm was utilized to simulate the propagation of reflected and transmitted beams within the forest canopy. Our method accurately modeled the solar radiant fluxes and demonstrated good agreement (*R*^2^ ≥ 0.82) with the plot-scale results of hemispherical photo-based HPEval software and pyranometer measurements. The maximum incident radiant flux appeared in the conifer plot at noon on June 15 due to the largest solar altitude angle (81.21°) and dense clustering of tree crowns; the conifer plot also received the maximum reflected radiant flux (10.91-324.65 kW) due to the higher reflectance of coniferous trees and the better absorption of reflected solar beams. However, the broadleaf plot received more transmitted radiant flux (37.7-226.71 kW) for the trees in the shaded area due to the larger transmittance of broadleaf species. Our method can directly simulate the detailed plot-scale distribution of canopy radiation and is valuable for researching light-dependent biophysiological processes.

## 1. Introduction

Shortwave solar radiation (wavelengths of 0.3-3 *μ*m) contains approximately 97.5% of the energy of solar radiation (wavelengths of 0.1-10 *μ*m) [1]. Shortwave radiation is therefore associated exclusively with the daytime at a particular location on the Earth's surface and hence is an important source of energy for plant physiological processes, such as photosynthesis and transpiration [2]. However, the shortwave solar radiation received by the plant canopy is dynamic not only due to the incident solar intensity and direction but also due to the canopy structure. Obtaining the shortwave radiative flux of a plant canopy is a frontier research field in forest phenotyping [3] and is highly important for the selection and breeding of highly light-efficiency tree architectures and for guiding the forest management of productive stands [4]. Solar radiation regimes also affect net primary productivity, forest species abundances, ecosystem response mechanisms, and site adaptation traits [5].

Prior knowledge of the local and regional solar radiation received by horizontal surfaces is essential for properly measuring the solar radiation of a target forest canopy. Some solar radiation models, e.g., the American Society of Heating, Refrigerating and Air-Conditioning Engineers (ASHRAE) algorithm [6] and the Iqbal model [7], can calculate the corresponding magnitude based on many readily measured quantities, such as the type and coverage of clouds, the atmospheric turbidity, the perceptible water content, and the sunshine duration (SD). These models are widely used by engineering and architectural communities to predict the average daily global radiation with hours of sunshine. Some empirical functions in these models, e.g., the Ångström–Prescott [8], third-degree [9], and logarithmic [10] equations, have been coupled with existing pyranometer sensors to measure the solar radiation flux density at a specific site. Moreover, accurately representing the canopy light profile and the canopy radiation transmission model is important for predicting ecological dynamics and forest productivity and has thus attracted extensive attention from various research communities [11]. Hence, many methods and instruments have been created or produced to calculate the transmission of solar radiation through the canopy according to the principle of modern optical imaging and advanced computer techniques, which may be grouped into three broad categories: hemispherical photograph-based (DHP) methods, other solar radiation instruments for field measurements, and ray-tracing-based methods.

DHP methods [12, 13] employ an upward-looking fisheye lens that takes forest photos with different zenith angle ring compositions, which records the plant vegetative distribution and the sky visibility through canopy crevices or gaps to derive the solar radiation transmission gap fraction or the leaf area index (LAI) for the obstruction of conducting light. Various commercial and noncommercial software programs [12, 14] are available for hemispherical photo (HP) processing and analysis; these programs can be used in combination with existing solar radiation equations to calculate the amount of radiation within the photo mapping area. Embedding a DHP method into an instrumental measurement is a relatively direct approach [15], and the measured radiation difference between the top (or open area with no overstory), vertical stratification, and bottom of the canopy can be used to evaluate the spatial solar irradiance distribution [16], light transmission profiles [17], and amount of intercepted radiation [13] among the canopies. Similarly, other handheld optical instruments and sensors have also been developed to estimate the transmission of solar radiation in field measurements for solar, agricultural, meteorological, and hydrological applications; such devices include the quantum sensor [18], which has a good quantum response and can assess the photosynthetic photon flux density under the plant canopy in a forest plot, the pyranometer [19], which measures the global solar radiation from a solid angle of 2*π* radians into a plane, the LAI-2200 [20], which is equipped with an LAI-2250 optical sensor and measure the transmission of radiation underneath the canopy based on the readings above the canopy to provide a reference, and the ceptometer [21], which consists of linear arrays of light sensors connected in parallel on a long probe to determine the vertical light interception of row crops. These instruments can be deployed in the field to obtain spatially sampled results of the vertical stratification of the solar radiation intercepted by plant canopies within a research area. In contrast, ray-tracing-based methods [22, 23] simulate the transfer of radiation within plant canopies and employ some assumptions or simplifications to approximate the canopy architecture; these techniques can be coupled with various computer graphics algorithms [24] of ray tracing methods to simulate the propagation and interception of light [25] or photons [26] to determine the trajectories of all light beams and photons within the canopy while synchronously considering the algorithm's efficiency [27]. In contrast, laser scanning techniques are capable of reflecting the detailed tree crown properties and depicting the geometrical characteristics of target tree crowns [28]. The Beer–Lambert law [29] calculates the probability that a beam is intercepted or attenuated as a mathematical function and is always used in tandem with both terrestrial [23] and airborne [30] laser scanning techniques that rely on countable emitted and received echoes to compute the solar transmittance and to simulate radiative transfer among forests.

Despite the numerous approaches and instruments proposed by researchers to calculate canopy radiation, many challenges continue to undermine existing methodological frameworks. For example, DHP methods and other instruments impose high requirements on the accuracy of the measurement processes and the surrounding environmental conditions [31]. Cameras with fish eye lenses and other instruments must always be absolutely horizontal beneath the forest canopy. The results of the DHP method inside the canopy suffer from unexpected deviations caused by partial shadows, uncertain fraction calculations stemming from varying camera exposure values of the camera device parameters, and the presence of dust or water vapor in the air, all of which introduce noise into the images. Under inhomogeneous lighting conditions or gaps, the LAI-2200 is always affected by problematic sunlit leaves, which requires the use of a view cap to eliminate the detrimental factors of unequal sky conditions (clouds or clearings), and leads to increases in the operational complexity and subjective selection variants [32]. In addition, the systematic errors incurred in other instruments, such as pyranometers or quantum sensors, are due to deviations of the embedded versatile sensors in terms of the particular spectral sensitivity and efficiency and depend on various types of radiation [18]. Moreover, for large forest stands, inconsistences exist between the high spatiotemporal sampling resolution, the rigidly gridded device measurements in woodland plots, and the statistical analyses used to quantify the solar radiation received by the forest, reducing the generalizability of the DHP and instrument methodologies, and experimental work requires extensive sampling data and many readings, which are time-consuming and laborious. In contrast, ray-tracing-based methods simulate the transmission, reflection, and incidence of solar beams within tree crowns and require a considerably large number of rays to adequately sample each small vegetative element in the canopy [33]. Accordingly, the computational complexity of ray-tracing-based methods has been shown to be exponentially correlated with the level of geometrical detail of heterogeneous tree crown models and the number of emitted solar rays in the target scenario [27]. Hence, a compromise between the level of phenotypic traits of the crown and the computational cost must be reached when simulating the physiological processes of a plant system at the detailed organ level [34]. An alternative option, namely, utilizing airborne light detection and ranging (LiDAR) scanning along with the Beer–Lambert law, has been implemented to substitute solar beams to determine the laser penetration index according to actual forest scenarios and realize a local insolation radiation transfer simulation [35]. However, airborne LiDAR data cannot be used directly to meticulously depict the vertical multilayer forest structure variables due to occlusion effects, leading to an inadequate account of the incident and transmitted solar radiation at the individual stand scale, a restrictive portrayal of the results from the given incident beam angles which are fixed in the absence of variability estimation across time [36], and the various inconsistencies that arise between the laser footprint size and the neighborhood search size for single backscattered echoes [30].

To overcome the shortcomings of the existing methods for calculating solar radiation, our approach, which approaches this problem from another perspective, utilizes the points collected by airborne LiDAR to reveal the spatial distributions and properties of the crown at the stand scale with the assistance of individual tree crown segmentation algorithms [37]. Consequently, many key parameters of each tree crown can be derived, such as the crown width, tree height, clear bole height, and position, which enable a forest plot to be simplified into an array of pseudoturbid canopy media using regular geometric primitives. Moreover, with the synergetic use of computer graphics techniques, solar radiation models, and airborne LiDAR technologies, crown surfaces can be triangulated, ray tracing tasks can be performed by simulating shortwave forcing at the study site, and locally shiny tree areas illuminated by simulated sunrays can be identified. The proposed method overcomes the complications associated with the fine characterization of tree organs and the occlusion of sunlight in plant systems by organ-scale heterogeneity and is convenient for assessing the 3D spatiotemporal variations of shortwave radiation in forest plots without requiring the repetitive collection of airborne LiDAR data at different angles.

The main objective of this paper is to calculate the shortwave radiative flux of a forest canopy by ascertaining the emission and collision of solar beams with quantifiable energy in many spatial triangles covering each tree surface composing the forest canopy, making it possible to retrieve the amount of incident shortwave radiation (wavelengths of 0.3-3 *μ*m) absorbed by the green parts of the illuminated tree crowns and the reflected and transmitted shortwave radiation components absorbed by the neighboring and shaded tree crowns in lower light conditions. Finally, the distribution of shortwave radiation received by a forest stand is intuitively visualized with the obtained quantitative results, which are validated by the existing DHP method and instrumental field measurements to verify the efficacy of the proposed approach.

## 2. Materials and Methods

### 2.1. Study Site and LiDAR Data

**Figure 1:**
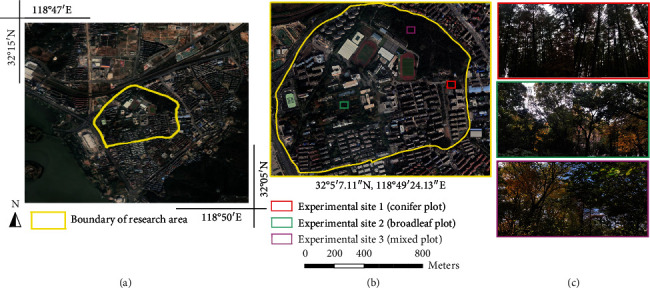
General situation of the study site. (a) The location of the study site, i.e., Nanjing Forestry University, Nanjing, Jiangsu Province, China (Google Earth). (b) Magnified view of the study site, where the three squares in different colors mark the boundaries of the different experimental plots. (c) Corresponding photos of the stands growing in the three experimental sites.

**Figure 2:**
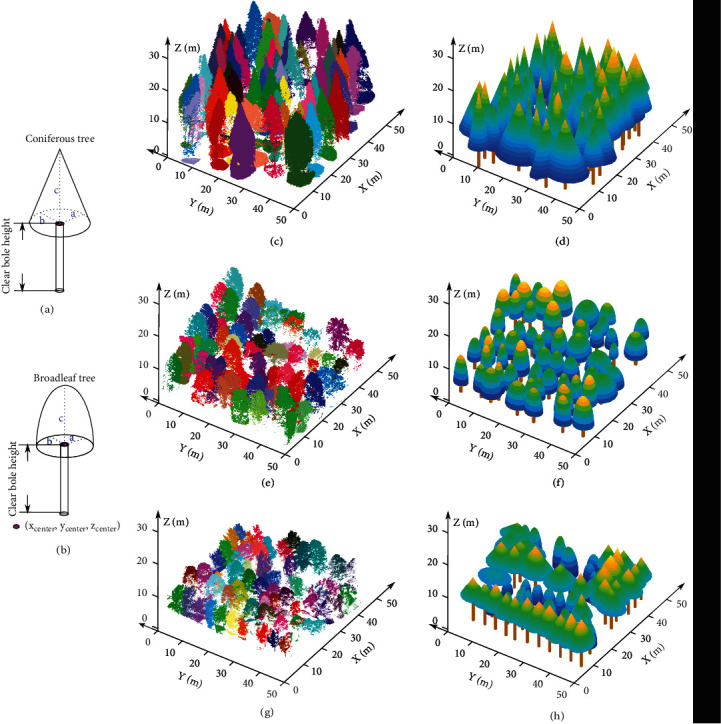
Individual tree crown segmentation from the airborne LiDAR data (c, e, and g) of the three experimental plots and 3D forest plot scenario reconstructions (d, f, and h) using basic geometric models with the calculated sizes according to the stand growth attributes retrieved from LiDAR data. (a and b) Cone and semiellipsoid models with variable parameters to simulate each conifer and broadleaf tree crown, respectively. (c and d) Pure Metasequoia conifer plot. (e and f) Broadleaf tree plot comprising multiple tree species. (g and h) Mixed species plot.

The study site is located on the Nanjing Forestry University campus in Nanjing (32°04′34.53^″^N, 118°48′42.06^″^E), southeastern Jiangsu Province (Figure 1). As a National Forest City, Nanjing has a warm and humid subtropical monsoon climate with four distinct seasons and abundant rainfall. The average annual precipitation here is 1000 to 1600 mm, and the average annual temperature is 15.4°C. Nanjing Forestry University covers a total area of 0.838 km^2^ and reaches a maximum elevation of 43 m. The tree population in this area consists of 7 main tree species, including Metasequoia (Metasequoia glyptostroboides H. H. Hu and W. C. Cheng), Chinese fir (Cunninghamia lanceolata (Lamb.) Hook.), cedar (Cedrus deodara (Roxb.) G. Don), ginkgo (Ginkgo biloba L.), Sapindus (Sapindus mukorossi Gaertn.), poplar (Populus L.), and camphor (Cinnamomum camphora (L.) J. Presl). As shown in Figure 2, three experimental site types on the campus were chosen for our experiments: experimental site 1 was a pure coniferous forest comprising M. glyptostroboides; experimental site 2 was a broadleaf forest composed of many tree species, e.g., Liriodendron chinense, Ulmus pumila, and Chinese ash; and experimental site 3 was a mixed broadleaf-conifer forest with a variety of mixed tree species, e.g., C. camphora, sweet osmanthus, Japanese cherry, Picea asperata mast, and Cedrus deodara. Three subsets, one from each of the three forest types (sites), with a plot size of 50 m × 50 m were chosen as test sites in the following experiments; the locations and photos of the test sites are shown in the middle and right columns of Figure 1.

LiDAR data for the study plots were obtained on May 20, 2019, using a Velodyne HDL-32E laser scanner (produced by Velodyne Lidar Inc. headquartered in San Jose, CA, USA) flown 100 m above ground level with a flight speed of 10 km/h and a flight line overlap of 30%. The scanner emits a wavelength of 930 nm at a pulse repetition frequency of 21.7 kHz with a vertical field of view (FOV) of -30.67°–10.67° and a horizontal FOV of 360°. The beam divergence was 2.79 mrad. The average ground point distance was 10.5 cm, and the pulse density was approximately 100 points/m^2^. Figure 2 shows the point clouds collected from the three experimental plots at the study site.

### 2.2. Shortwave Solar Irradiance Calculation for the Study Site

The Iqbal model [7] was adopted in this study to evaluate the shortwave solar irradiance *I*_*total*_ of the horizontal surfaces at the study site. *I*_*total*_ include the direct irradiance *I*_*direct*_ originating from the Sun, the diffuse ground-level irradiance *I*_*diffuse*_ composed of Rayleigh scattering, aerosol scattering, and the multiple reflections between the ground and sky:

(1) $$\begin{array}{c} I_{total}=I_{direct}+I_{diffuse}, \end{array}$$

where *I*_*direct*_ = 0.9751*I*_*sc*_*r*_0_*τ*_*r*_*τ*_*o*_*τ*_*g*_*τ*_*w*_*τ*_*a*_sin*θ*_*a*_; the factor 0.9751 is included because the spectral interval considered here is 0.3-3 *μ*m; *I*_*sc*_ is the solar constant, which can be taken as 1367 W/m^2^; and *r*_0_ is the eccentricity correlation factor of the Earth's orbit. *τ*_*r*_, *τ*_*o*_, *τ*_*g*_, *τ*_*w*_, and *τ*_*a*_ are the Rayleigh, ozone, gas, water, and aerosol scattering transmittances, respectively. The expression for calculating the solar altitude angle *θ*_*a*_ is as follows:

(2) $$\begin{array}{c} \sin\theta_{a}=\sin\theta_{l}\sin\theta_{d}+\cos\theta_{l}\cos\theta_{d}\cos\theta_{h}, \end{array}$$

where *θ*_*l*_ is the local latitude, *θ*_*d*_ is the current declination angle of the Sun, and *θ*_*h*_ is the hour angle in local solar time.

The diffuse irradiance reaching the horizontal plane can be expressed as

(3) $$\begin{array}{c} I_{diffuse}=I_{dr}+I_{da}+I_{dm}, \end{array}$$

where *I*_*dr*_ and *I*_*da*_ represent the Rayleigh-scattered and aerosol-scattered diffuse irradiance arriving on a horizontal ground surface, respectively [38], and *I*_*dm*_ describes the multiple reflections between the ground and sky. *I*_*dr*_ can be determined as

(4) $$\begin{array}{c} I_{dr}=\frac{I_{sc}r_{0}\tau_{o}\tau_{g}\tau_{w}\tau_{aa}\left(1-\tau_{r}\right)\sin\theta_{a}}{2\left(1-m_{a}+m_{a}^{1.02}\right)}, \end{array}$$

where *τ*_*aa*_ is the transmittance of direct radiation due to aerosol absorptance and is given by *τ*_*aa*_ = 1‐(1‐*ω*_*o*_)(1‐*m*_*a*_ + *m*_*a*_^1.02^)(1‐*τ*_*a*_), where *ω*_*o*_ is the single-scattering albedo fraction of incident energy scattered to the total attenuation by aerosols and is taken to be 0.9. *m*_*a*_ is the air mass at the actual pressure.

The diffuse irradiance caused by aerosols is calculated as follows:

(5) $$\begin{array}{c} I_{da}=\frac{0.79I_{sc}r_{0}\tau_{o}\tau_{g}\tau_{w}\tau_{aa}F_{c}\left(1-\tau_{as}\right)\sin\theta_{a}}{1-m_{a}+m_{a}^{1.02}}, \end{array}$$

where *F*_*c*_ is the fraction of forward scattering relative to total scattering and is taken to be 0.84. The chosen values of 0.79 represent soot-containing (urban) aerosols that are different from sulfate-like (rural) aerosols [39]. *τ*_*as*_ is the fraction of the incident energy transmitted after aerosol scattering and is given by

(6) $$\begin{array}{c} \tau_{as}=\frac{\tau_{a}}{\tau_{aa}}. \end{array}$$

The diffuse irradiance produced by multiple reflections between the ground and the sky can be determined as

(7) $$\begin{array}{c} I_{dm}=\frac{\left(I_{direct}+I_{dr}+I_{da}\right)\left(\rho_{g}\rho_{a}\right)}{1-\rho_{g}\rho_{a}}, \end{array}$$

where *ρ*_*g*_ is the ground albedo and *ρ*_*a*_ is the albedo of the cloudless sky and can be computed as

(8) $$\begin{array}{c} \rho_{a}=0.0685+\left(1-F_{c}\right)\left(1-\tau_{as}\right). \end{array}$$

Through the above process, the shortwave solar irradiance of the study site can be evaluated.

### 2.3. Tree Crown Simulation Using Regular Geometric Primitives

After the airborne LiDAR data of real forest stands on our campus were collected, the scanned data corresponding to each of the three experimental plot types were refined to remove noise and outliers [40]. Then, each treetop was detected using a dual Gaussian filter, and individual crown segmentation was conducted based on the energy function minimization approach [37]. In this way, each experimental plot was decomposed into a collection of individual trees, thereby facilitating the retrieval of the growth properties for each tree, including the crown width in two perpendicular directions (north–south and east–west), tree height, tree center coordinates, and clear bole height. In addition, a forest survey was conducted by the authors as an auxiliary source of data for ascertaining the species and quantities of trees in the three experimental plots, which served as references to optimize the calculated results by our algorithms.

The forest stands studied in the three experimental plots can be divided into broadleaf trees and coniferous trees. Geometric primitives, namely, semiellipsoids and cones, were employed to simulate broadleaf and coniferous tree crowns, respectively, and were assigned adaptive parameters according to the growth properties of each separate tree crown retrieved from the point clouds. formulas (9) and (10) show the spatial semiellipsoid and cone formulas for different broadleaf and coniferous tree crown simulations, respectively:

(9) $$\begin{array}{c} \frac{{\left(x-x_{center}\right)}^{2}}{a^{2}}+\frac{{\left(y-y_{center}\right)}^{2}}{b^{2}}+\frac{{\left(z-z_{center}\right)}^{2}}{c^{2}}=1\;z\in \left[z_{center},{height}_{tree}\right], \end{array}$$

(10) $$\begin{array}{c} \frac{{\left(x-x_{center}\right)}^{2}}{a^{2}}+\frac{{\left(y-y_{center}\right)}^{2}}{b^{2}}-\frac{{\left({height}_{tree}-z_{center}\right)}^{2}}{c^{2}}=0\;z\in \left[z_{center},{height}_{tree}\right], \end{array}$$

where *a* and *b* represent the half-crown width of each individual tree in the east–west and north–south directions, respectively; *c* is the tree height minus the clear bole height; the coordinates of each crown center of the segmented tree are recorded as (*x*_*center*_, *y*_*center*_, *z*_*center*_); and *z*_*center*_ is the clear bole height of the corresponding tree. The schematic representations for modeling the tree crowns using geometric primitives are shown in Figures 2(a) and 2(b).

Using our method for the three selected experimental plots yielded 60, 60, and 61 individual segmented trees in plots 1, 2, and 3, respectively. Each individual tree was then specified with its spatially explicit coordinates and growth parameters (shown in Table 1), which were adopted to adjust the semiellipsoid and cone models to represent each tree crown. The three plot scenarios displayed using the substituted geometric model compositions are shown in Figures 2(d), 2(f), and 2(h).

### 2.4. Triangulation of the Tree Models

**Figure 3:**
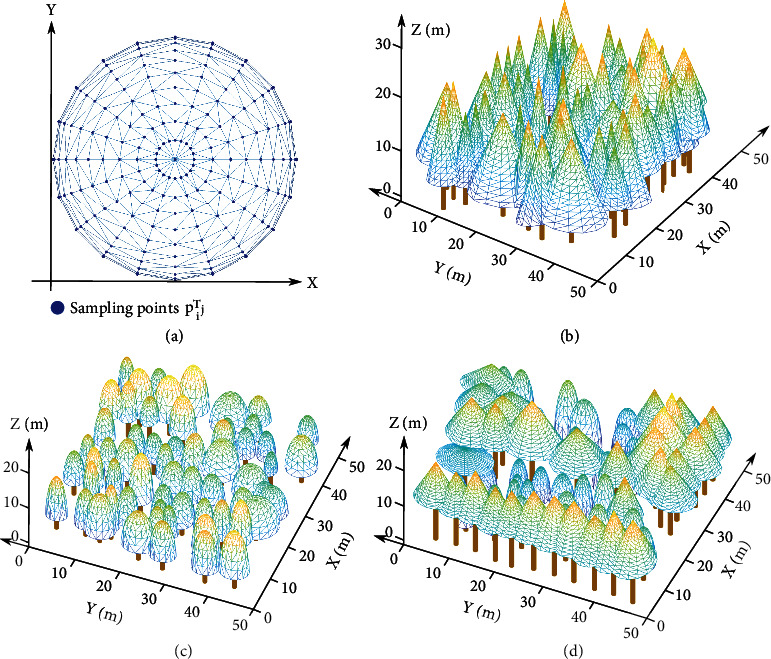
Triangulated forest canopy surfaces composed of many geometric primitives substituting for various tree crowns. (a) Sampling points were distributed on each tree crown and projected onto the *X*–*Y* plane to optimize the triangulation. (b–d) The triangulation results for the simulated geometric primitives representing each tree crown constituting the three experimental plots.

After every tree crown in the plot was represented by geometric primitives, triangulation was performed to decompose all tree crown surfaces into a set of triangles *T*_*j*_, *j* = 1, 2, ⋯*M* with explicit descriptions at the triangle scale. This process ensures that the tree crowns were adequately sampled with triangles covering the exterior surface of the forest canopy and optimally illustrates the detailed interplay of solar beam transmission and reflection among tree crowns. Because 3D Delaunay triangulation is excessively complex and computationally expensive, 2D Delaunay triangulation was adopted here to reduce the program execution time. First, vertical projection of the geometric surfaces of the cone and semiellipsoid representing every tree crown was conducted to form the projected circles on the *X* − *Y* plane, and many concentric circles with equal distances inside each projected circle were defined (Figure 3). Then, *ξ* points on each concentric circle with equal arc length were taken to obtain the sampling points *p*_*i*_^*T*_*j*_^ on the *X* − *Y* plane. Second, the 2D Delaunay triangulation algorithm was employed to generate a triangle mesh based on these sampling points (Figure 3(a)), and the index of each sampling point *p*_*i*_^*T*_*j*_^ defining the vertex of each triangle composing the tree crown surface was obtained. Finally, according to the sampling point indices, a reverse projection was carried out to realize 3D Delaunay triangulation for the surface of each tree crown. This strategy obviously improved the efficiency of the program and made 3D triangulation unnecessary for our tree models. The triangulated tree models are visualized in Figure 3.

### 2.5. Discretization of Solar Radiation

Shortwave solar radiation on the forest canopy is based on the interaction between solar beams and forest surfaces and can define the propagation of solar light within forest plots to quantitatively assess the incidence, transmission, and reflection of solar beams. To simulate the solar radiation energy from the Sun at infinity, the solar lights emitted from evenly distributed source points on a horizontal plane over the experimental plots in the same direction were simulated with a defined interval between neighboring source points, where the coordinates of the source points are denoted (*x*_*k*_^*source*^, *y*_*k*_^*source*^, *z*_*k*_^*source*^), *k* = 1, 2, ⋯, *N*, where *N* represents the total number of source points, and one solar beam was emitted from each source point. Here, the fixed interval between two neighboring points was set as 0.2 m; i.e., the density of emitted solar beams was *ℓ* = 25/*m*^2^.

The heights of the trees among the three experimental plots ranged from 9 to 31 m; thus, we set the magnitude of *z*_*k*_^*source*^ for all source points, i.e., the height of the horizontal plane over the experimental plots on which the source points were located, to 100 m to simulate the downward propagation of sunlight onto the forest canopy. Additionally, the horizontal plane (800 × 800 m) is much larger than the experimental plot (50 × 50 m) below, which guarantees that the emitted solar beam from the source points with varying solar elevation and azimuth angles can fully cover the target experimental plot below and illuminate the trees on the boundaries of each plot.

The direction of each solar beam from each source point was represented as the unit vector ${\overset{⟶}{L}}_{k}\left(v_{k,x}^{light},v_{k,y}^{light},v_{k,z}^{light}\right)$, which is correlated with the current solar azimuth angle $\theta_{z}=\arccos\left(v_{k,y}^{light}/\left(\sqrt{{\left(v_{k,x}^{light}\right)}^{2}+{\left(v_{k,y}^{light}\right)}^{2}}\right)\right)$ and altitude angle $\theta_{a}=\arcsin\left(v_{k,z}^{light}/\left(\sqrt{{\left(v_{k,x}^{light}\right)}^{2}+{\left(v_{k,y}^{light}\right)}^{2}+{\left(v_{k,z}^{light}\right)}^{2}}\right)\right)$. Hence, when the orientation and source point are known, a simulated solar light beam *L*_*k*_ can be expressed as follows:

(11) $$\begin{array}{c} L_{k}:\frac{x-x_{k}^{source}}{v_{k,x}^{light}}=\frac{y-y_{k}^{source}}{v_{k,y}^{light}}=\frac{z-z_{k}^{source}}{v_{k,z}^{light}}. \end{array}$$

Because the density of incident solar beams is *ℓ* = 25/*m*^2^ and the calculated shortwave solar irradiance *I*_*total*_ (W/m^2^) of the study site mentioned in subsection 2.2 is known, the radiant power or radiant flux of each emitted solar beam can be expressed as *I*_*total*_/*ℓ*.

#### 2.5.1. Determination of the Local Tree Crown Area Exposed to Radiation

When solar light hits the canopy, the local area of the tree canopy exposed to incident light that conducts photosynthesis must be determined. Here, with the generated triangles *T*_*j*_ with the three corresponding vertices *p*_1_^*T*_*j*_^, *p*_2_^*T*_*j*_^, and *p*_3_^*T*_*j*_^ and the simulated solar light beams *L*_*k*_, a beam intersection algorithm can be adopted to determine the locally illuminated and shaded areas of the simulated tree crowns at the triangle scale.

The normal vector of each triangle *T*_*j*_ can be calculated based on its three vertices *p*_*l*_^*T*_*j*_^(*p*_*l*,*x*_^*T*_*j*_^, *p*_*l*,*y*_^*T*_*j*_^, *p*_*l*,*z*_^*T*_*j*_^), *l* = 1, 2, 3:

(12) $$\begin{array}{c} {\overset{⟶}{N}}^{T_{j}}\left(N_{x}^{T_{j}},N_{y}^{T_{j}},N_{z}^{T_{j}}\right)=\left(p_{2,x}^{T_{j}}-p_{1,x}^{T_{j}},p_{2,y}^{T_{j}}-p_{1,y}^{T_{j}},p_{2,z}^{T_{j}}-p_{1,z}^{T_{j}}\right)\times \left(p_{3,x}^{T_{j}}-p_{1,x}^{T_{j}},p_{3,y}^{T_{j}}-p_{1,y}^{T_{j}},p_{3,z}^{T_{j}}-p_{1,z}^{T_{j}}\right), \end{array}$$

where *x* represents the cross product of two vectors. Combined with the incident light formula (11), formula (13) was adopted here to ascertain whether the line *L*_*k*_ intersects with the plane in which triangle *T*_*j*_ lies.

(13) $$\begin{array}{c} F=N_{x}^{T_{j}}v_{k,x}^{light}+N_{y}^{T_{j}}v_{k,y}^{light}+N_{z}^{T_{j}}v_{k,z}^{light}. \end{array}$$

If *F* = 0, the emitted solar light is parallel to the plane of the triangle and does not intersect the plane. If *F* ≠ 0, an intersection point exists. The coordinates of the intersection point *p*^*inte*^(*p*_*x*_^*inte*^, *p*_*y*_^*inte*^, *p*_*z*_^*inte*^) are

(14) $$\begin{array}{c} \begin{array}{c} p_{x}^{inte}=\frac{x_{k}^{source}\left(N_{y}^{T_{j}}v_{k,y}^{light}+N_{z}^{T_{j}}v_{k,z}^{light}\right)+v_{k,x}^{light}\left(N_{x}^{T_{j}}p_{1,x}^{T_{j}}+N_{y}^{T_{j}}\left(p_{2,x}^{T_{j}}-y_{k}^{source}\right)+N_{z}^{T_{j}}\left(p_{3,x}^{T_{j}}-z_{k}^{source}\right)\right)}{F},\\ p_{y}^{inte}=\frac{y_{k}^{source}\left(N_{x}^{T_{j}}v_{k,x}^{light}+N_{z}^{T_{j}}v_{k,z}^{light}\right)+v_{k,y}^{light}\left(N_{y}^{T_{j}}p_{2,x}^{T_{j}}+N_{x}^{T_{j}}\left(p_{1,x}^{T_{j}}-x_{k}^{source}\right)+N_{z}^{T_{j}}\left(p_{3,x}^{T_{j}}-z_{k}^{source}\right)\right)}{F},\\ p_{z}^{inte}=\frac{z_{k}^{source}\left(N_{x}^{T_{j}}v_{k,x}^{light}+N_{y}^{T_{j}}v_{k,y}^{light}\right)+v_{k,z}^{light}\left(N_{z}^{T_{j}}{xp}_{3}^{T_{j}}+N_{x}^{T_{j}}\left(p_{1,x}^{T_{j}}-x_{k}^{source}\right)+N_{y}^{T_{j}}\left(p_{2,x}^{T_{j}}-y_{k}^{source}\right)\right)}{F}. \end{array} \end{array}$$

After calculating the intersection point *p*^*inte*^(*p*_*x*_^*inte*^, *p*_*y*_^*inte*^, *p*_*z*_^*inte*^), the following criteria are adopted to determine whether the intersection point is inside triangle *T*_*j*_:

(15) $$\begin{array}{c} \begin{array}{c} \left(N_{1}\times N_{2}\right)\cdot \left(N_{2}\times N_{3}\right)>0,\\ \left(N_{1}\times N_{2}\right)\cdot \left(N_{1}\times N_{3}\right)>0,\\ \left(N_{2}\times N_{3}\right)\cdot \left(N_{1}\times N_{3}\right)>0, \end{array} \end{array}$$

where *N*_1_, *N*_2_, and *N*_3_ are derived by

(16) $$\begin{array}{c} \begin{array}{c} N_{1}=\left(p_{x}^{inte}-p_{1,x}^{T_{j}},p_{y}^{inte}-p_{1,y}^{T_{j}},p_{z}^{inte}-p_{1,z}^{T_{j}}\right),\\ N_{2}=\left(p_{x}^{inte}-p_{2,x}^{T_{j}},p_{y}^{inte}-p_{2,y}^{T_{j}},p_{z}^{inte}-p_{2,z}^{T_{j}}\right),\\ N_{3}=\left(p_{x}^{inte}-p_{3,x}^{T_{j}},p_{y}^{inte}-p_{3,y}^{T_{j}},p_{z}^{inte}-p_{3,z}^{T_{j}}\right). \end{array} \end{array}$$

**Figure 4:**
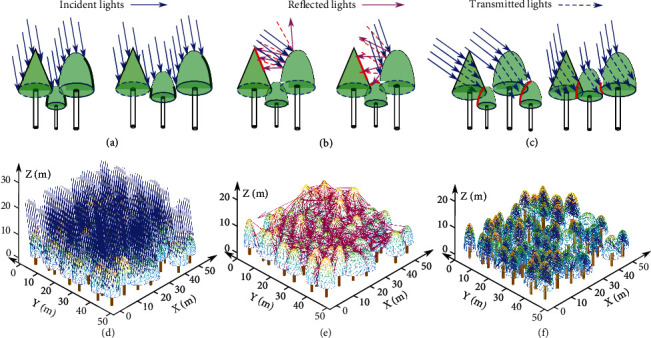
Schematic representation depicting solar ray tracing among the experimental plots. (a) Incident solar beams irradiating neighboring trees with occluded areas labeled by dark green curves. (b) A large fraction of incident solar beams with a lower altitude angle are reflected towards the adjacent tree crowns, while solar beams with a higher altitude angle are reflected towards the suppressed trees in the mid and understory layers. The red lines represent the area illuminated by the reflected and transmitted solar beams. (c) The transmitted solar rays at varying azimuth angles penetrate the tree crowns that first encounter with the decay energy illuminating the trees behind the front tree crowns along the ray path. Incident (d), reflected (e), and transmitted (f) beams were decimated to portray the propagation of solar light among the experimental plots.

Through the above calculation, the intersection of each solar beam with each triangle facet covering the forest canopy can be determined. Due to the mutual occlusion effect of vegetative elements throughout the forest, each beam is considered to be intercepted by the first triangle facet that the beam encountered. Hence, the triangle-scale area of the forest canopy illuminated by direct solar irradiance at different solar angles can be determined. This resolves the problem of overlap between heterogeneous tree crowns within a complex forest canopy. The schematic representation and decimated incident solar beams are shown in Figure 4, where the dark green curves mark the mutually occluded areas at the current incident angle of the solar beams, and the red curves mark the area illuminated by reflected and transmitted solar beams.

#### 2.5.2. Incident Radiant Flux Calculation

After determining the triangles that are irradiated, we need to further quantify the incident radiant flux of each illuminated triangle covering the forest canopy and then extrapolate the incident radiant flux of the entire forest stand.

Based on the above canopy light simulation, the first intersection between incident solar light beams and triangles can be determined, as shown in Figures 4(a) and 4(b). Suppose that the total number of first intersection points in every triangle *T*_*j*_ with the emitted solar light beams is *num*_*T*_*j*__^*inte*,*incident*^, which means that a total of *num*_*T*_*j*__^*inte*,*incident*^ emitted light beams are first intercepted by the triangles. Then, the incident radiant flux or incident radiant power of trees in the experimental plot can be expressed by the following formula:

(17) $$\begin{array}{c} E_{incident}=\overset{{num}_{T_{j}}^{inte,incident}}{\underset{d=1}{\sum }}\frac{I_{total}}{\ell }\cos\left(\theta_{{\overset{⟶}{L}}_{d}}^{T_{j}}\right), \end{array}$$

where $\theta_{{\overset{⟶}{L}}_{d}}^{T_{j}}=\arccos\left(\left({\overset{⟶}{N}}^{T_{j}}\cdot {\overset{⟶}{L}}_{d}\right)/\left(\left|{\overset{⟶}{N}}^{T_{j}}\right|\left|{\overset{⟶}{L}}_{d}\right|\right)\right)$ is the included angle between the incident solar light beam *L*_*d*_ and the normal vector of the irradiated triangle *T*_*j*_ and *I*_*total*_/*ℓ* represents the aforementioned radiant flux of each solar beam.

#### 2.5.3. Reflected Radiant Flux Calculation

Reflection is more complicated than incidence. According to the law of reflection in geometric optics [41], the specular reflection of the angle at which the light is incident on the surface equals that of the angle at which it is reflected. The reflection vector (*v*_*d*,*x*_^*reflect*^, *v*_*d*,*y*_^*reflect*^, *v*_*d*,*z*_^*reflect*^)of the incident solar beams *L*_*d*_ reflected by different triangles *T*_*j*_ of the forest canopy can be derived by the following formula:

(18) $$\begin{array}{c} {\overset{⟵}{L}}_{d}:\left(v_{d,x}^{reflect},v_{d,y}^{reflect},v_{d,z}^{reflect}\right)={\overset{⟶}{L}}_{d}-2\left({\overset{⟶}{L}}_{d}\cdot {\overset{⟶}{N}}^{T_{j}}\right){\overset{⟶}{N}}^{T_{j}}. \end{array}$$

After calculating the reflection vector for the reflected solar light beams irradiating the forest canopy triangles, we assume that a number of reflected solar beams *num*_*T*_*j*__^*inte*,*reflect*^ with reflective energy continue to propagate at a given reflection angle and interact for a second time with other triangles *T*_*j*_ of the forest canopy. Then, the reflected radiant flux among the trees in the experimental plot can be expressed by the following formula:

(19) $$\begin{array}{c} E_{reflect}=\overset{{num}_{T_{j}}^{inte,reflect}}{\underset{q=1}{\sum }}\frac{I_{total}}{\ell }\cos\left(\theta_{{\overset{⟵}{L}}_{q}}^{T_{j}}\right)\rho_{reflect}, \end{array}$$

(20) $$\begin{array}{c} \theta_{{\overset{⟵}{L}}_{q}}^{T_{j}}=\arccos\left(\frac{{\overset{⟶}{N}}^{T_{j}}\cdot {\overset{⟵}{L}}_{q}}{\left|{\overset{⟶}{N}}^{T_{j}}\right|\left|{\overset{⟵}{L}}_{q}\right|}\right). \end{array}$$

Formula (19) determines the points within the triangles that intersect with the reflected solar light beams, where *ρ*_*reflect*_ is the canopy reflectance of various tree species to which the triangle facets belong. According to the measured spectral properties of leaves and needles, the average reflectance values of broadleaf and coniferous trees for shortwave radiation in the wavelength range from 0.3 to 3 *μ*m were set as 0.17 and 0.25, respectively [42, 43]; $\theta_{{\overset{⟵}{L}}_{q}}^{T_{j}}$ is the included angle between the reflected solar light beam and the normal vector of the triangle; and *q* is a temporal variable representing every incident solar light beam after the reflection generated by a second collision with the triangular forest canopy elements. The reflected lights yielding various local reflections among the forest canopy are shown in Figures 4(b) and 4(e). When the incident solar light beams have smaller altitude angles, Figure 4(b) shows that the reflected ray trajectories mainly collide with adjacent canopy trees. While the solar altitude angles are larger, the reflected rays yield anisotropic directions that illuminate the various forest floors of the vicinity, including the canopy and understory layers, which constructs a real simulation process for reflected radiation transport in complex forest habitats.

#### 2.5.4. Transmitted Radiant Flux Calculation

When incident solar light beams with the given solar altitude angle strike the tree crowns, part of the light energy is reflected and absorbed by vegetative elements in the tree crowns, whereas the rest of the beam energy penetrates the tree crown and hits the trees behind the front tree crowns. Regarding the inside of the canopy and the outside of the canopy as two different media, the transmitted beams entering the canopy can be seen as refracted beams. Here, refraction was simulated following Snell's law [44]; i.e., for a given pair of media, the ratio of the sines of the angle of incidence *θ*_1_ and angle of refraction *θ*_2_ is equal to the refractive index between the two media. According to the law of refraction in geometric optics, the vector ${\overset{⟶}{L}}_{d}^{trans}\left(v_{d,1}^{trans},v_{d,2}^{trans},v_{d,3}^{trans}\right)$ can be derived by the following formula:

(21) $$\begin{array}{c} {\overset{⟶}{L}}_{d}^{trans}\left(v_{d,x}^{trans},v_{d,y}^{trans},v_{d,z}^{trans}\right)=\frac{{\overset{⟶}{L}}_{d}}{e}+\left(\frac{-{\overset{⟶}{N}}^{T_{j}}\cdot {\overset{⟶}{L}}_{d}}{e}-\sqrt{1-\frac{1-{\left(-{\overset{⟶}{N}}^{T_{j}}\cdot {\overset{⟶}{L}}_{d}\right)}^{2}}{e^{2}}}\right){\overset{⟶}{N}}^{T_{j}}, \end{array}$$

where *e* represents the refraction index and is taken as 0.5 in this study.

The calculation equation for the transmitted radiant flux for the trees behind the irradiated tree crown by the incident solar beams in the target plot is as follows:

(22) $$\begin{array}{c} E_{trans}=\overset{{num}_{T_{j}}^{inte,trans}}{\underset{d=1}{\sum }}\frac{I_{total}}{\ell }\cos\left(\theta_{{\overset{⟶}{L}}_{d}^{trans}}^{T_{j}}\right)\tau_{trans}, \end{array}$$

(23) $$\begin{array}{c} \theta_{{\overset{⟶}{L}}_{d}^{trans}}^{T_{j}}=\arccos\left(\frac{{\overset{⟶}{N}}^{T_{j}}\cdot {\overset{⟶}{L}}_{d}^{trans}}{\left|{\overset{⟶}{N}}^{T_{j}}\right|\left|{\overset{⟶}{L}}_{d}^{trans}\right|}\right). \end{array}$$

Formula (22) calculates the points of the triangles that intersect only the transmitted solar light beams, where *nump*_*T*_*j*__^*inte*,*trans*^ is the total number of first intersections between all the transmitted (refracted) solar beams and a collection of triangles *T*_*j*_ and *τ*_*trans*_ is the canopy transmittance pertaining to the related triangles of various tree species. According to the measured optical properties of leaves and needles, the average transmittance values of broadleaf and coniferous trees for shortwave radiation in the wavelength range from 0.3 to 3 *μ*m were set as 0.2 and 0.15, respectively [42, 45], and $\theta_{{\overset{⟶}{L}}_{d}^{trans}}^{T_{j}}$ is the included angle between the transmitted (refracted) solar light beam and the normal vector of the triangle irradiated by the beam. As illustrated in Figures 4(c) and 4(f), the incident solar light beams penetrate the tree crown with attenuated light energy, and the transmitted light beams illuminate the subsequent tree crowns obstructed by the front trees, which is similar to the actual case in which light beams pass through the gaps or translucent media of tree crowns modeled by the Beer–Lambert law [46] with diluted energy reaching the subcanopy layers of existing shade-tolerant species therein.

### 2.6. Verification Methods

To further verify the effectiveness of our method, two existing methods, i.e., HPEval software for calculating the transmission of shortwave radiation in a canopy based on high-resolution HP [14] and a solar radiation sensor (RS-RA-N01-AL) to measure the solar power per unit area, were employed to calculate the solar radiation intercepted by a forest canopy; the results were compared with those of our method.

HPEval is a software tool written in the MATLAB programming language designed to evaluate digital HPs and provide local shortwave radiation estimates. Intended for applications below the forest canopy, this tool can output high-resolution time series and has the potential to resolve individual sunflecks. By default, HPEval outputs potential shortwave radiation with the corresponding above-canopy radiation, but it can also calculate the true solar radiation intercepted by the canopy. The entire procedure includes image acquisition, metadata collection, image binarization, and sky pixel segmentation. The HP input to HPEval was taken by a high-resolution digital camera (Canon EOS 5D Mark III) with a fisheye lens (Canon EF 8-15 mm). The camera was placed on the ground surface within the experimental plot with the optical axis pointing upwards to take the HPs of the crown. To allow the image to intersect with the astronomical path parameters of the Sun, the camera must be horizontally level and oriented towards the north. The resulting photos record the structure of the plant canopy and the portion of the sky that is visible through holes or crevices in the canopy. These features can be accurately measured and used by HPEval software to calculate the solar radiation transmitted through (or intercepted by) the plant canopy.

**Figure 5:**
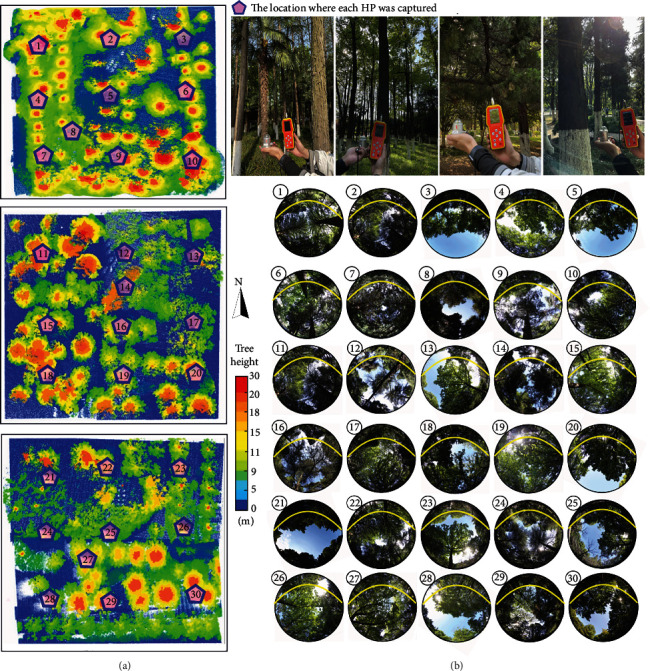
Shortwave radiation measurements for the three experimental plots using a pyranometer (a) and DHP HPEval software (b). The left color bar indicates the variations in tree heights of the three experimental plots, and the light pink pentagons represent the locations where HPs were taken. Nine HPs were captured for each plot with an approximate spacing of 12.5 m, and another captured HP at a separate location as complementary data. The yellow curves in HPs represent the path of the Sun on the day of image acquisition.

Figure 5 shows that 10 HPs were processed for each plot. The adjacent HPs are separated by a fixed interval of 12.5 m, and the light pink pentagons represent the locations where HPs were taken. We used the following formula to calculate the solar shortwave radiation intercepted by the trees in the plot *S*_*intercept*_:

(24) $$\begin{array}{c} S_{intercept}=S_{above}-S_{below}=S_{above}-\left(S_{dif}\cdot V_{f}+S_{dir}\cdot \tau_{dir}\right), \end{array}$$

where *S*_*above*_ is the incident above-canopy solar (shortwave) radiation equal to the calculated instantaneous shortwave solar irradiance mentioned in subsection 2.2 and *S*_*below*_ is the incident below-canopy solar shortwave radiation composed of diffuse *S*_*dif*_ and direct *S*_*dir*_ components, which can be derived from the solar altitude angle at that time and the solar constant [47]. Each HP was split into many zenith angle rings, and a weighted average of the ratios of sky pixels to all pixels for these rings was calculated as *V*_*f*_. *τ*_*dir*_ is the transmissivity of direct shortwave radiation, which is referred to here as the ratio of the pixels classified as sky to the total number of pixels in each HP.

The area of each plot (50 m × 50 m) was 2500 m^2^. Hence, the average incident radiant flux of trees in each plot calculated from the HPs using HPEval software can be expressed as the following formula:

(25) $$\begin{array}{c} E_{incident}=\overset{10}{\underset{i=1}{\sum }}S_{intercept}^{i}\cdot \left(\frac{2500}{10}\right). \end{array}$$

The instrumental measurement method shown in Figure 5 adopted a pyranometer that incorporates a photoelectric solar total radiation sensor (RS-RA-N01-AL) that measures the solar energy received from the entire hemisphere (180° field of view). The sensor was produced by Shandong Renke Control Technology Co., Ltd. It adopts the photoelectric principle and can be used to measure the total radiation value under sunlight (wavelengths of 0.3-3 *μ*m). The sensor adopts a high-precision photosensitive element that has high and wide spectral absorption in the full spectral range and sound stability; the product adopts the standard Modbus-Remote Terminal Unit (RTU) 485 communication protocol and connects with a handheld data recorder to read the current solar radiation value. According to the locations of the center points of the sampling HPs in each plot, the solar radiation above the canopy *I*_*above*_ was represented by respective measurements from an open site near the plot, and the solar radiation below the canopy *I*_*below*_ was measured at another set of measurement positions close to the ground surface at the center points of the HPs. Utmost care was taken to ensure that both sets of radiation sensor measurements were horizontally level.

The measuring range of the pyranometer is 0~1800 W/m^2^. The specific calculation formula deriving the incident radiant flux for the trees in the experiment plot is as follows:

(26) $$\begin{array}{c} E_{incident}=\overset{10}{\underset{i=1}{\sum }}\left(I_{above}-I_{below}\right)*\left(\frac{2500}{10}\right). \end{array}$$

## 3. Results

### 3.1. Shortwave Solar Irradiance Calculation for the Study Site

Through the method for calculating the shortwave solar irradiance in a local area elaborated in subsection 2.2 of this article in combination with the longitude, latitude, cumulative days, and angle of altitude, as well as the corresponding air quality parameters, the shortwave solar irradiance of the study site on our campus at every moment was calculated.

**Figure 6:**
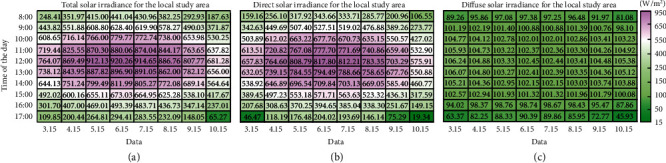
Calculated shortwave solar irradiance of the study site on different dates and at different times of day. The three plots present the calculated total (a), direct (b), and diffuse (c) solar irradiance on the horizontal ground surface of the study site.

At different times of day, the exposure situations of the experimental plots under solar irradiation differed. The solar altitude and azimuth angles vary with time, thereby affecting the shortwave solar irradiance of the study site. For example, in the Northern Hemisphere, the solar altitude angle at noon on the summer solstice is obviously higher than the solar altitude angle at noon on the winter solstice. Based on the Iqbal model described in subsection 2.2, the 15th of each month from March to October 2019 was selected, and the hourly shortwave solar irradiance on the horizontal ground surface from 8 : 00 to 17 : 00 was calculated with a one-hour interval under clear-sky conditions; the results are shown in Figure 6. In the morning and afternoon, the Sun is lower in the sky, and thus, its rays travel farther through the atmosphere, decreasing the amount of shortwave solar irradiance. Similarly, during the summer season (from June to August), the shortwave solar irradiance is larger when the solar altitude angle is higher than that during other months at the same time of day.

### 3.2. Illumination Rendering Results for the Three Plots with the Retrieved Radiant Flux

**Figure 7:**
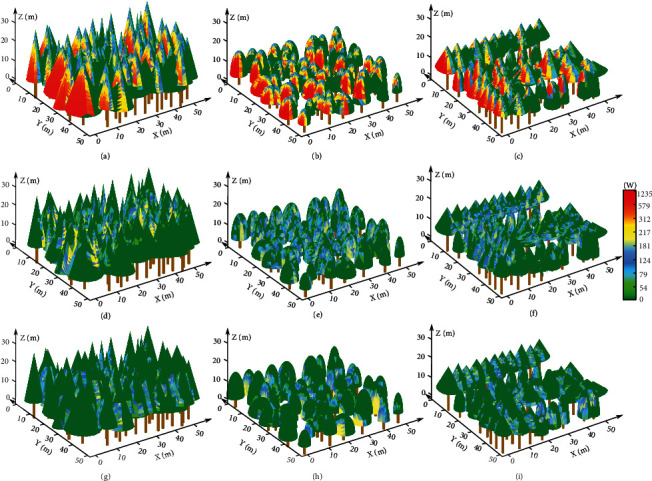
Canopy radiant flux distributions within the conifer plot, broadleaf plot, and mixed tree species plot at 11 : 00 on July 15, 2019. Changes in the color from green to red indicate an increase in the magnitude of radiant flux received locally by the triangles composing the forest canopy. The leftmost column depicts the calculated distributions of incident (a), reflected (d), and transmitted (g) radiant fluxes of the conifer plot. (b, e, and h) Equivalent figures for the broadleaf plot. (c, f, and i) Equivalent figures for the mixed tree species plot.

The total forest canopy volumes for the conifer, broadleaf, and mixed tree species plots, i.e., the sum of geometrical primitives representing the volume of every individual tree crown in each plot, were 25151, 13546, and 15391 m^3^, respectively. The anisotropic forest architecture and heterogeneous stem structures affect the spatial distribution of incident solar light beams among the stands in the plots; for example, the mutual occlusion between tree crowns caused by the varying geometric structure of the canopy generates local shadows, which further affects the propagation trajectories of the reflected and transmitted beams among the forest stands based on the ray tracing algorithm.  Figure 7 shows the incident, reflected, and transmitted beams intercepted by the triangles composing the forest canopies of the three sample plots at 10 : 00 on July 15, 2019, when solar light was incident from the upper left side of the plot with a solar azimuth angle of 102.32° and an altitude angle of 61.58°.

In other words, Figure 7 illustrates the distributions of the different lighting hierarchies at the triangle scale, where solar light beams illuminate trees locally and propagate among forest stands according to the ray tracing algorithm through a geometric representation of the forest canopy.

For incident solar light with oblique illumination, the incident radiant flux is concentrated on the side of the forest canopy facing the prevailing Sun, while the opposite side has a lower radiant flux and a number of shaded areas. In addition, the mutual occlusions caused by large trees affect the solar illumination of the surrounding smaller trees. Under a sparse radiance distribution, the reflected radiant flux emerges mainly in the middle and lower layers of the forest stands. Solar light enters forest stands via transmission and refraction through forest canopy gaps or vegetative elements. The simulated distributions of beams interacting with the tree crowns in the shaded area behind the trees under incident illumination were adopted to calculate the radiant flux of transmitted light within the forest canopy.

**Figure 8:**
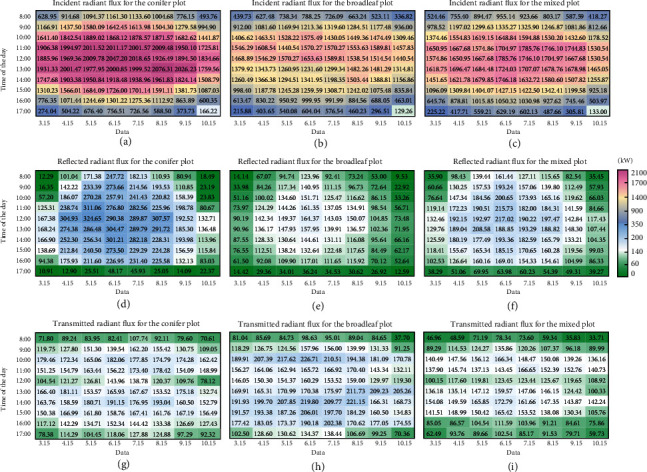
Radiant fluxes of the conifer, broadleaf, and mixed tree species plots calculated with the date and time of day as variables. (a–c) The incident radiant fluxes of the conifer plot, broadleaf plot, and mixed species plot, respectively; (d–f) the reflected radiant fluxes of the conifer plot, broadleaf plot, and mixed tree plot, respectively; (g–i) the transmitted radiant fluxes of the conifer plot, broadleaf plot, and mixed species plot, respectively.

Regardless of the compositions of the homogeneous forest stands, Figure 8 illustrates that the incident radiant flux peaks at approximately noon. Since the azimuth angle at noon in the Northern Hemisphere is always 180° (south), the solar altitude angle at noon is an important factor in different months (ranging from March 15 to October 15). As shown in Figure 8, at noon, the solar altitude angle in Nanjing City reached its peak on June 15 (81.21°), with a total shortwave solar irradiance of 920.26 W/m^2^ for the study site. Therefore, the incident radiant fluxes at noon on June 15 (2047.2 kW for the conifer plot, 1653.63 kW for the broadleaf plot, and 1785.76 kW for the mixed plot) were higher than the radiant fluxes at noon in the other months (ranging from 1834.66 kW in October to 2018.65 kW in July for the conifer plot, from 1440.54 kW in March to 1589.81 kW in July for the broadleaf plot, and from 1530.54 kW in October to 1746.1 kW in July for the mixed plot). In addition, all the trees grow vertically with spatially outspread tree crowns due to plant phototaxis and tree-tree competition; hence, the high altitude angle at which the Sun emits solar light is aligned with the tree growth directions and therefore eliminates the overlapping regions between tree crowns in the forest stands regardless of the absorption of light by the subcanopy (understory) vegetation. However, as the Sun's altitude angle decreases, e.g., in the early morning and late afternoon, oblique solar beams with small solar altitude angles (near 15°) are always obstructed by trees in front, leading to considerable decreases in the incident radiant fluxes of the forest stands and reaching the minimum range of 129.26–756.51 kW at 17 : 00. Combined with the tree growth attributes listed in Table 1, as shown in Figures 8(a)–8(c), the incident radiant flux is the largest for the conifer plot, where the highest coniferous trees with relatively large crowns result in a tightly packed forest canopy and a larger interception area for obliquely propagating sunlight. In contrast, the pure broadleaf plot receives the smallest incident radiant flux due to the shortest mature trees and relatively small crowns, thereby yielding more open space within the forest landscape.

The reflected and transmitted radiant fluxes are correlated with the number of reflected and transmitted light beams that recollide after the collision between the incident laser beams and geometric primitives of each tree crown. A number of reflected light beams with new orientations possibly propagate either to shrubs in the understory layer or outside the experimental plot without radiative transport to the nearby stands. Similarly, partial incident light beams penetrate through the illuminated tree crowns, with the remaining energy being absorbed by the triangles on the surface of the tree crowns located behind the illuminated tree crowns. As mentioned in subsection 2.5, the assigned canopy reflectance of coniferous trees (0.25) is larger than that of broadleaf trees (0.17), but the transmittance of coniferous trees (0.15) is less than that of broadleaf trees (0.20). In conjunction with the included angle between the reflected or transmitted light beam and the surface of the target for another interaction, the reflected radiant flux was greater than the transmitted radiant flux for the coniferous tree plot, while the reverse was true for the broadleaf tree plot. In contrast, the mixed tree species plot exhibited roughly equal numbers of coniferous (33) and broadleaf (28) trees, but conifers have a larger tree crown than do broadleaf trees, driving the mechanism of space occupancy and potential light competition at the tree level; hence, the assessed transmitted radiant flux was smaller than the reflected radiant flux. Figures 8(d)–8(f) show that the maximum reflected radiant fluxes of 324.65 kW, 164.37 kW, and 217.02 kW were derived for the conifer, broadleaf, and mixed plots, respectively, at noon, when the solar altitude was at its peak with maximum individual solar beam energy. Figures 8(g)–8(i) show that when the solar altitude angle was approximately 65° at 10 : 00 or 14 : 00, the maximum transmitted radiant fluxes were 193.04 kW for the conifer plot, 226.71 kW for the broadleaf plot, and 172.79 kW for the mixed plot, which are attributed mainly to the transmitted solar beams emitted from the inclined topside that increase the probability of hitting the second tree crown along the propagation path but are also influenced by many factors, e.g., the transmittance of different tree species, the degree to which the stems are clustered, the crown surface area, and the morphological structures, as shown in Figures 7(g)–7(i).

**Figure 9:**
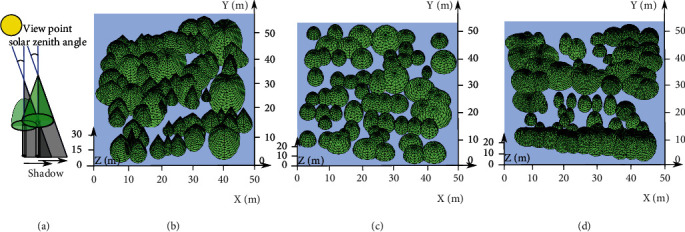
Perspectives of the three forest stands parallel to the direction of the solar incident beams at noon on August 15, 2019, with a solar altitude angle of 73.01° and a solar azimuth angle of 180.00°. Light blue represents the ground, and the canopy is represented by the composition of spatial green triangular patches.

Variations in the azimuth angle were accompanied by changes in the solar altitude angle, offering more possibilities for changes in the produced radiant flux and different illuminated fields of view of the target plot than are usually anticipated [48].  Figure 9(a) is a schematic representation showing that the solar light-illuminated area of the current plot is related to the density of trees, the heights of the growing trees, the shapes of the tree crowns, and the solar altitude and azimuth angles at 12 : 00 on August 15, 2019. The perspectives corresponding to the incident solar light direction in the three observed plots are presented in Figures 9(b)–9(d), which indicate that the conifer plot (b) has a larger area for receiving incident solar light beams, followed by the mixed tree species plot (d) and the broadleaf plot (c) in descending order.

### 3.3. Comparison with Existing Methods

Table 2 shows the calculated radiant fluxes received by the experimental plot using the three methods, i.e., our method, DHP, and HPEval software and pyranometer instrument. The determination coefficients *R*^2^ between the three methods are all higher than 0.82. A reasonable explanation for the imperfect alignment with the biases between our method and the DHP software estimates and the instrument-based field measurements is that the distance and orientation of each sampling point in a plot need to be strictly standardized in the field. Improving the sampling point density increases the determination coefficients between any two methods, but deviations due to data perturbations stemming from manual operation and judgement errors tend to arise. For HPEval software, incorrectly setting the threshold for the binarization of HPs may cause the porosity to be overestimated or underestimated, and uniform distributions of the leaf reflectance and transmittance under illumination conditions of varying intensity do not appropriately reflect the actual physiological situation with varying image grey values. All these factors lead to certain deviations between the solar radiation fluxes calculated based on HPEval and the actual values. Moreover, to effectively prevent environmental factors from interfering with internal components, the instrument-measured values should be closer to the true values, but sensors require considerable manpower and preparation to operate during the measurement process, e.g., locating the sampling points and conducting an accredited calibration; it is also difficult to implement instrumental measurements under strong winds and spectral content conditions differing from sunlight. Consequently, with a limited number of instruments, the collection of field data at all the sampling points in a plot at the same time is unrealistic. Therefore, to acquire field measurements, the instrument would need to be carried rapidly to every sampling location while guaranteeing correct measurement acquisition in pursuit of decreasing shortwave solar irradiance variations over time. In addition, dust on the sensor degrades its accuracy, and the limit of battery life interrupts and restricts the continuous operation of the device.

## 4. Discussion

### 4.1. Perspectives of Calculating the Solar Radiation for a Forest

Conveniently achieving accurate estimates of the shortwave radiation illuminating a forest canopy is essential for studying both the biophysical processes between developmental stages in the plant life cycle and the utilization of solar energy. Many research communities have used ray tracing algorithms (computer techniques), multiecho full-waveform airborne LiDAR for canopy transmittance estimation, and the Beer–Lambert law related to light attenuation from scanned data at the voxel scale with the aim of calculating the solar radiation striking the target forest [49]. The proposed method employed computer graphics technology [50] and airborne LiDAR data to capture the fine-spatial-resolution 3D spatiotemporal distribution of shortwave radiation in forest canopies and overcome some existing restrictions of current methods. The adopted strategy leverages the merits of airborne LiDAR data reflecting tree growth properties for tree crown modelling and attempts to ameliorate the occlusion effects at the stand scale among the middle and lower layers of the forest canopy, which causes the solar radiation intercepted by forests to be overestimated when using the Beer–Lambert law [51]. A ray tracing model and basic translucent geometric models with the obtained crown sizes simulating every individual crown constituting the canopy architecture were employed to reproduce the realistic heterogeneity of light illumination and to estimate the radiant fluxes of various forest canopies. Changes in the date and time of day led to variations in the solar altitude and azimuth angles in conjunction with the spatially heterogeneous distributions of the tree crown size and density for various tree plots, which generated a variety of illumination perspectives and occlusion effects that changed the details of how the vegetation elements interacted with the solar beams emitted from the current position of the Sun in the sky. Moreover, the multifaceted phenomena related to light-driven plant growth were reflected by the extent of shading, and a mutually sheltered region of tree crowns and reflected light bouncing caused by plant morphogenesis were rendered.

We found that canopy packing and plot space filling increased the corresponding radiant flux magnitudes of each plot [52]. The total forest canopy volume of the conifer plot was larger than those of the broadleaf and mixed tree species plots, which is consistent with the largest incident radiant flux for the conifer plot. Although some studies [53, 54] elaborated that species composition is a significant factor that potentially impacts the pace of forest dynamics, the resilience of ecosystem productivity and the structural plasticity of the canopy, which improves the interception capability of solar radiation with dense photosynthetic tissues in the forest canopy layer that is more impenetrable for the solar beams. Meanwhile, tree morphological features, e.g., crown size and tree height, constrained by forest reforestation activities, silvicultural intervention intensities, and ecological microenvironment conditions, also influence the efficiency of plant photosynthesis [55]. Coniferous trees in the first plot were older and developed larger and deeper tree bodies with cone-shaped tree crowns, which increased the vertical stratification of the forest canopy, facilitating solar light covering the whole canopy [56]. Conversely, the broadleaf and mixed trees in the second and third plots had smaller tree crowns, diminishing the occupation of space and crown-to-crown interactions, leading to many solar light beams directly illuminating the ground without being fully utilized.

Tiny leaves irregularly distributed within tree crowns cause versatile specular and diffuse light reflections, which greatly complicate the calculations of the solar radiant flux among forest canopies [57]. Our approach exhibits comparatively weaker performance at extracting the correspondence between the radiant flux and distribution of numerous small leaves among tree crowns, but the proposed approach does not require a substantially large number of rays to adequately sample small vegetative elements and achieves a balance between the plot-level and species-level radiant flux calculations in conjunction with the LiDAR data-derived tree growth properties for the changing geometrical shapes of crowns among species. Furthermore, the reflected and transmitted beams originating from the solar beams with temporally varying directions of incidence that collide with the anisotropic surfaces of tree crowns are well predicted, and the trapping of transmitted light energy by trees growing in shaded areas is also reliably simulated. The whole framework depicts the incident illumination, reflected sunlight, and shaded stress tolerance for various forest stratification layers, all of which are valuable for exploring mixed species that are pursuing their physiological optimum conditions [58] and a harmonious coexistence [59]. Hence, compared to existing methods, our system is more practicable, scalable, and adaptable to any biome type and complex, structurally varied canopies and has implications for rendering the radiation received by many vegetation plot scenes.

### 4.2. Parameters of our Method and Algorithm Execution

The specific settings for the major parameters in our method are addressed as follows. In the process of modeling the tree canopy, we used the derived growth properties from airborne LiDAR data to model the forest canopy compositions according to the standard shape of conifers as cones and broadleaf trees as ellipsoids. Consequently, a triangulation strategy was utilized to subdivide the forest canopy into various detailed regions at triangle scales. Each discretized solar beam with the set energy calculated from the shortwave solar irradiance of the study site was emitted along the direction of the current solar altitude angle and zenith angle and then interacted with the triangles representing crown surfaces. In principle, the smaller the triangle and the greater the density of the incident beams, the more accurate the result of the forest radiant flux will be obtained, albeit with more program execution time.

**Figure 10:**
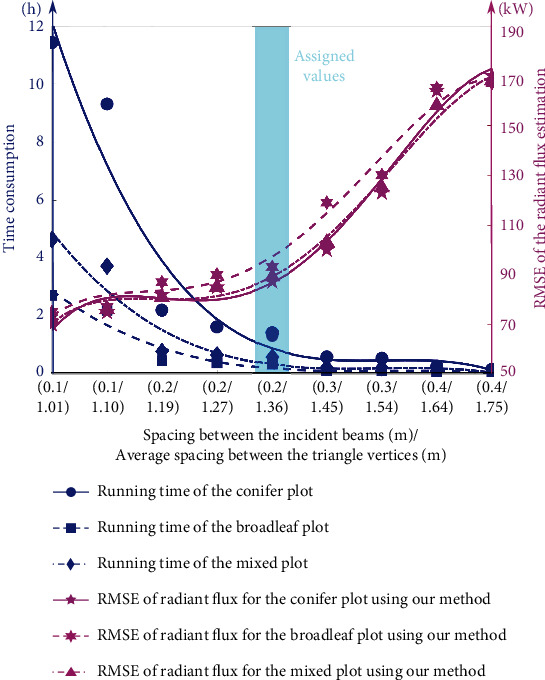
Dual *y*-axis plot showing the program running time and distribution of the calculated RMSE of radiant flux using our method against the DHP method and pyranometer measurements for conifer, broadleaf, and mixed tree species plots. Lines are fitted from cubic polynomial functions. The light blue areas represent the assigned parameter settings in our method comprehensively considering computational expense and RMSE magnitudes.

We changed the spacing between the neighboring incident solar beams (ranging from 0.1 m to 0.4 m) and the average spacing between the adjacent vertices of the triangles (ranging from 1.01 m to 1.75 m by tuning the number of concentric circles in the projected circle and number of sampling points on each concentric circle), which resulted in a greater number of incident solar beams ranging from 25236 to 402741 for the conifer plot with the tallest trees compared to the broader-leaf plot (ranging from 16310 to 265161) and mixed tree species plot (ranging from 19900 to 318003). Meanwhile, the number of triangles covering the conifer plot ranging from 14156 to 56755 was also larger than that of the broadleaf plot (ranging from 6705 to 20495) and mixed tree species plot (ranging from 9178 to 29159) due to taller trees with broader tree crowns in the conifer plot. A computer configured with Intel Core i7-9700K Desktop Processor 8 Cores up to 3.6 GHz in tandem with 32 GB RAM was employed for running the programs on the Microsoft Windows 8.1 operating system. All program codes in the work were written and executed in MATLAB (MathWorks, Inc., Natick, Massachusetts, USA). Since our laptop was not equipped with a graphics processing unit (GPU) for accelerating the creation of 3D forest models [60] and rendering of complex scene lighting, the average time consumption using our programs for the radiant flux calculation and graphical lighting rendering was relatively high. Therefore, the time required to calculate the intersections between solar beams and triangles on the forest canopies for the conifer plot (ranging from 0.18 h to 11.43 h with the density of incident beams and triangle vertices increasing) was higher than that for the mixed plot (ranging from 0.09 h to 4.64 h) and the broadleaf plot (ranging from 0.06 h to 2.72 h).  Figure 10 of the dual *y*-axis plot shows the variations in the root mean square error (RMSE) of radiant flux estimation and time consumption using our method for three experimental plots with the changing spacing of the incident beams and triangle vertexes. A steady upward RMSE trend of the estimated radiant flux between the results of our method and those of the reference field measurements (mean of the DHP HPEval and pyranometer instrument) for three experimental plots is represented by the pink solid and dashed lines obtained by cubic polynomial regression fitting. This trend illustrates that a dense light beam distribution and fine triangle representation can lead to a lower RMSE in the presence of nonirradiated triangles exist because a sparse light beam distribution will yield an insufficient sampling density. In contrast, the blue lines in Figure 10 show that the time consumption of our method for radiant flux calculation decreases and then plateaus as the spacing between neighboring incident beams or neighboring triangle vertices increases. Hence, a tradeoff of parameter settings with spacing between incident beams of 0.2 m and average spacing between triangle vertices of 1.36 m was decided, which was marked by a light blue area in Figure 10 under comprehensive consideration of the requirement of conserving average program execution time (0.78 h) and a relatively small average RMSE value (92.42 kW) for three plots.

In this study, we adopted empirical values of the reflectance and transmittance for broadleaf and conifer canopies instead of using the Beer–Lambert law for light attenuation [61] or airborne LiDAR data with a multiecho assessment [30] to calculate the forest canopy transmittance. One explanation is that the purpose of our method is to retrieve the diurnal and monthly variations in the shortwave radiation regimes at the plot scale considering the properties of the forest, e.g., the crown shape, the tree height, and the cluster degree. These variations can induce shielded effects and affect the availability of light in the canopy under varying incident solar angles, but the spatiotemporal variability of the forest canopy transmittance, which is impacted by plant phenological shifts and varying solar radiation angles, cannot be accurately assessed by the point clouds collected at a certain moment. Different locations and periods for emitted laser beams have varying observation perspectives with the acquired point clouds, which reflects the various morphologies of woodlands and can lead to distinct gap fraction estimations and variable plant physiological traits among the studied plots. In addition, many complications can impact current computer graphics techniques when portraying the vertical light transmittance profile in complex canopy architectures over time. For example, the geometrical development of fine scale descriptions of each vegetative element based on relatively sparse points acquired by the long-range scanning patterns of airborne LiDAR is challenging; the calculation of the total discrete light interception based on the leaf-scale heterogeneity with atypically discontinuous boundaries has a high computational complexity, and the occlusion metrics within the tree crown that lead to deficiencies in the scanned data when mapping vegetative material must be appropriately assessed. An abbreviated method [62] that substitutes the scanned points for crown materials and determines the probability of the points blocking incident solar beams is adopted with the intention of determining the crown transmittance under varying solar angles, which shows that the canopy transmittance mainly falls in the range of 0.2-0.5. However, due to the insufficiency of multitemporal scanned data, neglecting the solar beams passing through the spacing between neighboring points and a lack of GPU capacity to accelerate the modeling of numerous vegetative elements with an adequate amount of emitted ray collisions, hence, the optical properties at the tree species levels, e.g., the reflectance and transmittance of broadleaf and coniferous tree species, which have been studies previously [63, 64], were used herein. A pioneering study [65] showed that coniferous trees have higher reflectance values (ranging from 0.04 to 0.38) than broadleaf trees (ranging from 0.02 to 0.29) in the visible (VIS) spectrum (wavelengths from 380 nm to 750 nm). In the near-infrared (NIR) spectrum (wavelengths from 750 nm to 1400 nm), the reflectance value of coniferous trees range within 0.14–0.55, which is greater than those of broadleaf trees (ranging from 0.15 to 0.47). Only in the shortwave infrared (SWIR) spectrum (wavelengths from 1400 nm to 3000 nm) are the reflectances of coniferous trees (ranging from 0.02 to 0.28) slightly lower than those of broadleaf trees (ranging from 0.02 to 0.33). Regarding their spectral transmittance, values of coniferous trees (0.02–0.41) are higher than those of broadleaf trees (0.02–0.30) in the VIS spectrum, whereas the transmittance values of coniferous trees are definitely lower than those of broadleaf trees in the NIR (conifers: 0.05–0.32; broadleaves: 0.18–0.55) and SWIR spectra (conifers: 0.01–0.21; broadleaves: 0.03–0.51). Hence, in accordance with the records in the related literature [51, 66], reflectance values of 0.25 and 0.17 were assigned to coniferous trees and broadleaf trees, respectively, with corresponding transmittance values of 0.15 and 0.2. In contrast, it can be envisaged that jointly using hyperspectral and airborne LiDAR with segmented individual trees and recognized tree species, in combination with field surveys, will likely facilitate the explicit characterization of the optical properties of forest plot constituents in the future. However, sources of uncertainties, namely, variations in the biophysical traits of trees, interactions among crowns with overlapping local regions, and artificial forest interventions at the individual tree level that can lead to variations in the spatial layout of forests and phenotypic traits over time, need to be properly considered.

## 5. Conclusion

This study is based on a basic geometric representation of a 3D canopy structure for the purpose of developing an approach to quantitatively analyze the interception, reflection, and transmission of shortwave radiation received by a forest canopy. The forest canopy is modeled from airborne LiDAR data collected from the canopy, and the crown surfaces are subdivided into triangles, allowing the proposed method to quantify the incident, reflected, and transmitted solar radiant fluxes received by various forest canopy strata of different experimental plots through solar radiation models and computer graphics-based ray tracing algorithms. An optimized beam intersection algorithm that models forest canopies with geometric primitives is proposed to simulate the propagation of solar light through forest stands. Our results show that a conifer plot with a highly clustered distribution of trees featuring relatively large tree crowns will receive the greatest fluxes of incident and reflected irradiance. In contrast, broadleaf plots have relatively large transmitted radiant fluxes for the subcanopy trees due to their larger transmittance. Determination coefficients (>0.82) show that our results are consistent with those of the existing methods, namely, HPEval software and pyranometer instrument measurements. With the appropriate adjustments to the parameters, the proposed approach can be applied to any broadleaf or coniferous tree species and to any forest plot type. With advancements in laser scanning techniques, computer graphics algorithms and the performance of GPUs, our future work will focus on adjusting the light density as needed to infinitely approach the actual shortwave solar irradiance and meticulously modeling the vegetative elements in tree crowns based on point clouds to finely assess the biophysical and geometrical properties of forest scenarios and thereby render realistically natural phenomena of sunlight transport systems among forests.

## Figures and Tables

**Table 1:** Retrieved growth properties of the trees growing in the three plots from airborne LiDAR data using our individual tree crown segmentation algorithm.

	Number of trees (conifer/broadleaf)	Tree height (m)	Crown width (E–W)/(S–N) (m)	Average crown width (E–W)/(S–N) (m)	Clear bole height (m)
Plot 1	60 (60/0)	15.4–31.4	2.2–18.2/2.5–17.9	8.1/8.3	4.9–10.6
Plot 2	60 (0/60)	9.7–18.2	2.8–12.6/3.3–12.5	6.6/6.5	2.1–6.3
Plot 3	61 (33/28)	10.3–19.7	2.1–13.3/2.0–14.1	7.1/7.2	2.5–7.1

**Table 2:** Comparison of the radiant fluxes retrieved using our method with those obtained by DHP HPEval software and the pyranometer sensor.

	Radiant flux (kW) on June 15, 2019, at noon			Radiant flux (kW) on July 15, 2019, at noon			Radiant flux (kW) on august 15, 2019, at noon		
	Plot 1	Plot 2	Plot 3	Plot 1	Plot 2	Plot 3	Plot 1	Plot 2	Plot 3
Our method	2047.20	1653.63	1785.76	2018.65	1589.81	1746.10	1926.49	1538.54	1704.97
HPEval	2190.43	1693.81	1897.00	1936.74	1623.74	1884.33	1878.31	1478.37	1617.23
Instrument	2130.13	1718.01	1791.32	2061.87	1445.60	1858.96	1784.59	1404.11	1675.78
*R* ^2^	0.82 (O-H); 0.85 (O-I); 0.86 (H-I)								

Note: O-H: determination coefficient between our method and HPEval; O-I: determination coefficient between our method and the pyranometer; O-I: determination coefficient between HPEval and the pyranometer.

## Data Availability

All the MATLAB code used in this study has been uploaded to GitHub and made available at https://github.com/njyunting/Source-code-of-TingYun-Project-Shortwave/. The measured and calculated data in this study are available upon request by contacting the first and corresponding authors.
